# Placental deficiency of the (pro)renin receptor ((P)RR) reduces placental development and functional capacity

**DOI:** 10.3389/fcell.2023.1212898

**Published:** 2023-08-01

**Authors:** Lachlan G. Schofield, Richard G. S. Kahl, Samantha L. Rodrigues, Joshua J. Fisher, Saije K. Endacott, Sarah J. Delforce, Eugenie R. Lumbers, Jacinta H. Martin, Kirsty G. Pringle

**Affiliations:** ^1^ School of Biomedical Sciences and Pharmacy, College of Health, Medicine and Wellbeing, University of Newcastle, Callaghan, NSW, Australia; ^2^ Mothers and Babies Research Program, Hunter Medical Research Institute, New Lambton Heights, NSW, Australia; ^3^ School of Medicine and Public Health, College of Health, Medicine and Wellbeing, University of Newcastle, Callaghan, NSW, Australia; ^4^ School of Environmental and Life Sciences, College of Engineering, Science and Environment, University of Newcastle, Callaghan, NSW, Australia; ^5^ Infertility and Reproduction Research Program, Hunter Medical Research Institute, New Lambton Heights, NSW, Australia

**Keywords:** placenta, trophoblast (TC), prorenin receptor, placental development, blastocyst

## Abstract

The (pro)renin receptor ((P)RR; also known as *ATP6AP2*) is a multifunctional receptor. The (P)RR activates the tissue renin-angiotensin system (RAS) and is also involved in regulating integral intracellular pathways such as V-ATPase and Wnt/β-catenin signalling. Given this, the (P)RR may be associated with essential pathways in placentation, however its role within the context of pregnancy remains poorly characterised. The first trimester/extravillous trophoblast cell line, HTR-8/SVneo, underwent an siRNA knockdown where they were incubated for 24 h with a negative control siRNA or siRNA targeting *ATP6AP2* mRNA. xCELLigence real-time cell analysis was performed to assess the effect of *ATP6AP2* mRNA knockdown on HTR-8/SVneo cell proliferation, migration, and invasion. In subsequent experiments, GFP-encoding lentiviral packaged gene-constructs were used to knockdown (P)RR expression in the trophectoderm of C57/BL6/CBA-F1 mouse blastocysts. Blastocysts were incubated for 6 h with vehicle (no-virus), control virus (non-targeting shRNA and GFP), or (P)RR-knockdown virus ((P)RR shRNA and GFP) before transfer into recipient pseudo-pregnant Swiss CD1 female mice. Fetal and placental tissues were collected and assessed at embryonic age (EA) 10 and 18. (P)RR levels were measured in the labyrinth zone of day 18 placentae and stereological Merz grid analysis was performed to determine the volumetric distribution of trophoblasts, fetal capillaries, and the maternal blood space. We showed that a reduction of *ATP6AP2* expression in HTR-8/SVneo cells *in vitro,* impaired trophoblast proliferation, migration, and invasion. *In vivo,* decreasing placental labyrinth (P)RR expression adversely effected placental physiology, decreasing placental trophoblast number and total surface area available for exchange, while also increasing maternal blood space. Additionally, decreased (P)RR affected placental efficacy evident by the reduced fetal-placental weight ratio. Our study shows that the (P)RR is necessary for appropriate placental development and function.

## 1 Introduction

Appropriate placental development is essential for pregnancy success, with poor placentation underpinning all hypertensive pathologies of pregnancy (Furuya et al., 2008; Morrison, 2008). The (pro)renin receptor ((P)RR; also known as *ATP6AP2*) is expressed in the placenta throughout pregnancy, and is most abundant during the first trimester when most placental development occurs (Sihn et al., 2010). Additionally, the (P)RR is known to be aberrantly expressed in the placenta of women within pregnancy pathologies such as preeclampsia (Nartita et al., 2016). However, the functional role of (P)RR in placental development and pregnancy success remains unknown.

Although initially considered to only regulate the local renin-angiotensin system (RAS) (Nguyen et al., 2002), the (P)RR is now recognised as a multifunctional receptor (Morosin et al., 2021). In this regard, in addition to stimulating the RAS by activating renin and prorenin, the (P)RR is involved in regulating various other intracellular signalling pathways. For example, it is integral to pH regulation as a component of vacuolar-ATPase (V-ATPase), activates the wingless/integrated (Wnt)/ß-catenin pathway, and stimulates extracellular signal-regulated kinase 1 and 2 (ERK1/2)/mitogen-activated protein kinase (MAPK) signalling (Kinouchi et al., 2010; Matsuura et al., 2011). Thus the (P)RR exhibits significant physiological relevance in cellular homeostasis.

The truncated form of the (P)RR (known as the M8.9 segment) forms an essential part of the V-ATPase complex (Kinouchi et al., 2010), a proton pump regulating the cellular microenvironment by maintaining a pH gradient (Pamarthy et al., 2018). Ablation of the (P)RR (*ATP6AP2)* gene in mouse cardiomyocytes *in vivo*, resulted in reduced V-ATPase expression and subsequent vesicular acidification (Kinouchi et al., 2010). In bovine endometrial studies, the V-ATPase complex exhibits a shift from the apical to pericellular localisation in the luminal epithelium after implantation. This loss of polarity may reflect the requirement to reduce acidification of the fetal-embryonic environment (Skinner et al., 1999). Additionally, mouse embryos that are deficient in the V-ATPase c-subunit perish shortly after implantation (Sun-Wada et al., 2000). Collectively, these data suggest that dysregulation of *ATP6AP2* gene expression affects V-ATPase function and subsequently pregnancy progression.

Aside from its role as an accessory protein for the V-ATPase complex, the (P)RR is also involved in Wnt/β-catenin signalling. Here, the (P)RR acts as an adaptor between V-ATPase and the Wnt receptors; lipoprotein receptor-related protein 6 (LRP6) and Frizzled 8 (FZD8) (Ichihara and Yatabe, 2019). Additionally, V-ATPase facilitates acidification of LRP6, which is required for its phosphorylation. Phosphorylated LRP6 subsequently stabilises β-catenin, enabling Wnt receptor internalization and the subsequent transcription of Wnt target genes (Ichihara and Yatabe, 2019). Importantly, activation of the Wnt/β-catenin signalling pathway is known to play a supportive role in placental development, promoting invasive differentiation of human trophoblast cells *in vitro* (Pollheimer et al., 2006).

Binding of prorenin/renin to the (P)RR also promotes the phosphorylation of extracellular signal-regulated kinase 1/2 (ERK1/2), which is necessary for ERK1/2 regulation of transcription factors important for cellular proliferation, differentiation, apoptosis, and embryogenesis (Mebratu and Tesfaigzi, 2009). In support of this, knockdown of *ATP6AP2* gene expression significantly impairs (P)RR-mediated ERK activation in human vascular smooth muscle cells (Sakoda et al., 2007) demonstrating that the (P)RR plays an essential regulatory role in ERK1/2 signalling.

Although the (P)RR is well known to be important for cellular homeostasis and appears to have essential roles in embryogenesis and placentation, its specific role in placental development remains to be clarified. Thus, this study aimed to explore the role of (P)RR in a first trimester extravillous trophoblast cell line (HTR-8/SVneo) to determine if *ATP6AP2* gene expression is essential for trophoblast proliferation, migration, and invasion *in vitro*. We also extended our studies to examine the effects of placental *ATP6AP2* deficiency *in vivo* on placental and fetal development in a mouse model.

## 2 Materials and Methods

### 2.1 Ethics approval

#### 2.1.1 Cell line

Ethics approval was obtained from the University of Newcastle Human Research Ethics Committee (H-2020-0398) to carry out work using the HTR-8/SVneo cell line.

#### 2.1.2 Animal model

Ethics approval was obtained from the University of Newcastle animal research ethics committee (A-2017-713) to carry out this work. Experiments were compliant with the requirements of the Australian Code of Practice for the Care and Use of Animals for Scientific Purposes (National Health and Medical Research Council) (Council, 2013). Female C57/BL6/CBA-F1 mice aged 3–4 weeks, male C57/BL6/CBA-F1 mice aged 8–12 weeks, and female/male SWISS mice aged 8–10 weeks were obtained from the University of Newcastle animal resources facility. Female mice were housed in groups of 2-4 per cage and male mice were housed individually in a temperature-controlled facility (22–24°C) with 12/12 h (h) light/dark cycle. Rodent food pellets and water were available *ad libitum.*

### 2.2 HTR-8/SVneo cell culture

HTR-8/SVneo cells are immortalised first trimester trophoblast cells (*In Vitro* Technologies, North Park Australia). HTR-8/SVneo cells were cultured in complete RPMI-1640 media (Hyclone, Utah United States) supplemented with 10% fetal bovine serum (FBS; SAFC Biosciences, Kansas United States), 1% sterile-filtered L-glutamine (Gibco, California United States) and 1% antibiotic-antimycotic (Gibco) in a humidified incubator at 5% CO_2_ and 37°C.

### 2.3 Transfection of cells with ATP6AP2 siRNA

HTR-8/SVneo cells were seeded at a density of 2 × 10^5^ per well in 6-well plates with 2 mL of complete media without antibiotics and cultured at 37°C in 5% CO_2_ in air. 24 h after seeding, cells were transfected with 2.5 µL of lipofectamine 2000 transfection reagent (Invitrogen, Massachusetts United States) and an siRNA targeting the *ATP6AP2* gene (HSS115476, Invitrogen) to knockdown *ATP6AP2* mRNA expression (final concentration 5 nM) or negative (non-targeting) control siRNA (Invitrogen; final concentration 5 nM) in complete media containing Opti-MEM (Gibco) without antibiotics. Complete medium without antibiotics was replaced after 24 h. Cells and conditioned media were collected 48 h after transfection, snap frozen in liquid nitrogen and stored at −80°C until required.

### 2.4 xCELLigence Real-Time Cell Analyser (RTCA) proliferation, migration, and invasion assays

To monitor HTR-8/SVneo cell proliferation, migration, and invasion in real time, the xCELLigence Real-Time Cell Analyser (RTCA) dual purpose (DP) instrument (Agilent, California United States) was used. HTR-8/SVneo cells were transfected with *ATP6AP2* siRNA or negative control siRNA in flasks for 24 h (as described above) before undergoing trypsin digestion. They were then seeded onto either an E-plate to monitor proliferation or a cell invasion and migration (CIM)-plate to monitor migration and invasion. Cells were then incubated at 37°C in 5% CO_2_.

#### 2.4.1 Cell proliferation

Complete medium (without antibiotics) containing 1 × 10^4^ HTR-8/SVneo cells per 200 µL, was seeded into each well of the E-plate and allowed to attach for 24 h. Cell proliferation was then monitored for a further 48 h. Electrical impedance was recorded and converted to 30 min (min) epochs of cell index. The slope of the growth curve for each treatment was analysed over the 48 h period, following the initial 24 h cell plating incubation.

#### 2.4.2 Cell migration

A total of 160 µL of complete media (without antibiotics) was added to the lower chamber of the CIM-plate. The upper chamber was then attached and 40 µL of serum free medium was added to each well. The plate was incubated at 37°C for 1 h before addition of cells. Serum free medium containing 3 × 10^4^ HTR-8/SVneo cells per 100 μL, was seeded in each well and cell migration was monitored for 48 h. Electrical impedance was recorded, as the cells adhered to the electrodes under the porous membrane between the upper and lower chambers and converted to 15 min epochs of cell index. The slope of the migration curve for each treatment was analysed for 48 h after seeding.

#### 2.4.3 Cell invasion

Prior to seeding of cells, 30 µL of Matrigel (Corning, New York United States) was used to coat the base of the upper chamber of the CIM-plates. The Matrigel was allowed to set at 37°C for 5 h. Cell invasion assays were then performed using the same protocol as was used for the migration assay (see above), with the exception that cells were allowed to plate for 20 h and the slope of the invasion curve was analysed 48 h after cell plating.

### 2.5 C57/BL6/CBA-F1 mouse embryo collection and culture

**FIGURE 1:**
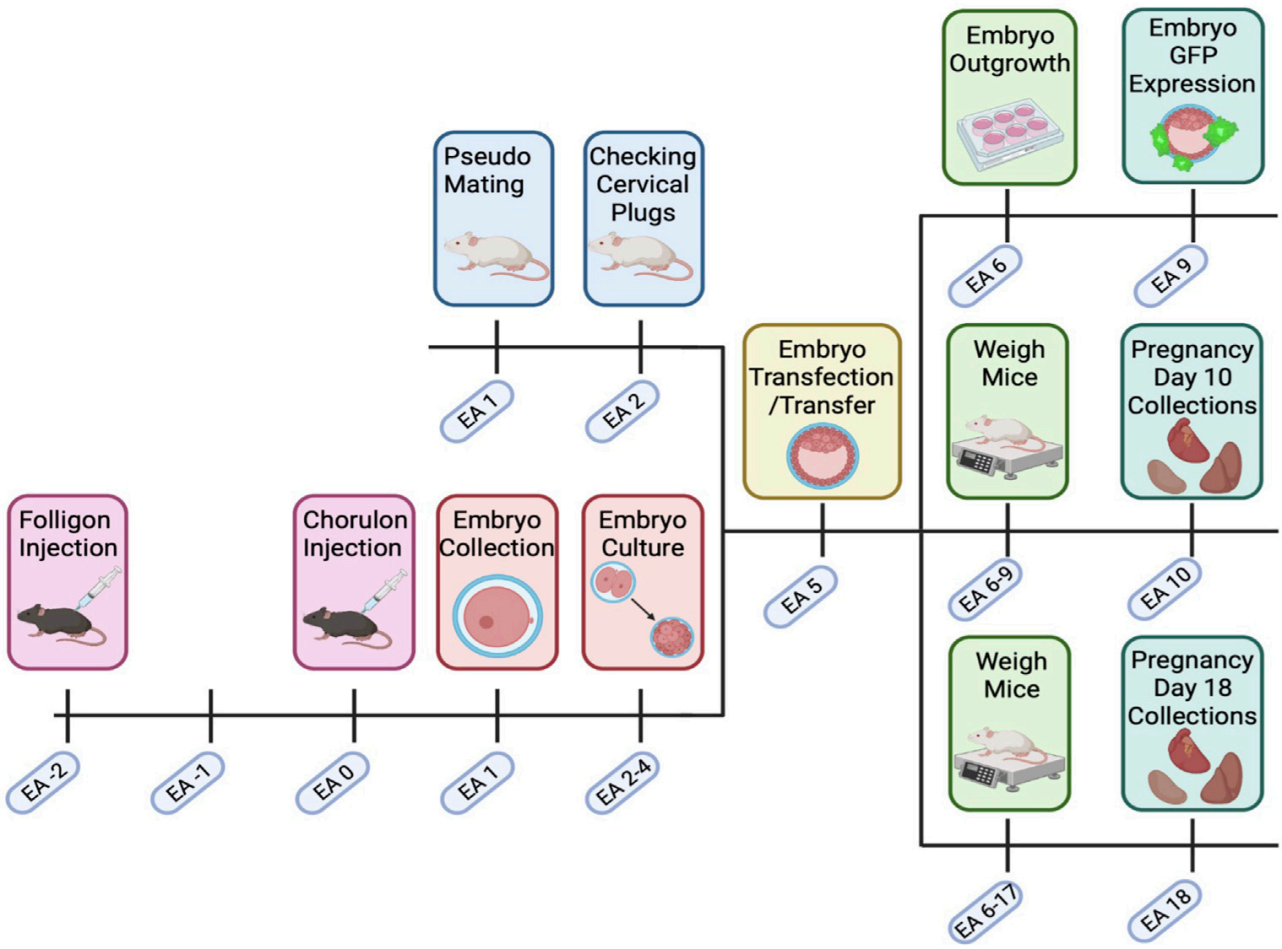
Transfected embryo mouse model timeline. C57BL/6. CBA (F1) mice were super ovulated with Folligon and Chorulon. F1 mice were mated, and presumptive zygotes were collected at embryonic age (EA) day 1. Embryos were then cultured until the blastocyst stage (EA 2–4) when they underwent zona pellucida removal and lentiviral transfection (EA 5). Blastocysts were then transferred into pseudopregnant recipient Swiss females. Pregnant mice were then weighed until EA 9 or EA 17, with fetal and maternal endpoint collections on EA 10 or EA 18. Excess embryos on EA 5 were cultured and assessed for GFP expression and outgrowth. Created with Biorender.com.

Superovulation was induced in 3-week-old female C57/BL6/CBA-F1 mice using timed intraperitoneal injections of Folligon (MSD Animal Health; 5IU/mouse, Bendigo AUS) at 16:00 h on embryonic age (EA) −2, followed by Chorulon (MSD Animal Health; 5IU/mouse) 48 h post initial injection (EA 0). Super ovulated females were then housed overnight with male C57/BL6/CBA-F1 mice for mating (2 females/male) as summarised in Figure 1. The following morning, females were euthanised by cervical dislocation and presumptive zygotes were collected from the oviducts and washed in G-1 plus media (Vitrolife, Gothenburg Sweden) (Martin et al., 2016; Martin et al., 2018; Martin et al., 2019). Presumptive zygotes were then treated with 300 μg/ml hyaluronic acid (Sigma-Aldrich, Missouri United States) for 30–45 s to remove the outer cumulus cells. Post hyaluronic acid treatment, presumptive zygotes were cultured in groups of 10 in 100 µL droplets of G-1 plus media and incubated at 37°C in 5% O_2_, 6% CO_2_, 89% N_2_. After incubation for 48 h, viable embryos were transferred into G-2 plus media (Vitrolife)(100 µL per 10 embryos) for 48 h and incubated as above. Throughout this process, culture media was covered with 1 ml Ovoil (Vitrolife). During culture, embryos were assessed for stage of growth and nonviable/arrested eggs and embryos (as described by Zhao et al. (Zhao et al., 2018)) were removed to reduce crossover effects.

### 2.6 Lentiviral infection of mouse blastocysts

At EA 5, cultured blastocysts were infected with GFP-encoding lentiviral gene constructs that either acted as a viral control or specifically knocked down *ATP6AP2* gene expression, as previously described by Chakraborty *et al* (Chakraborty et al., 2017). Briefly, blastocysts were exposed to Acid-Tyrode’s solution for 30 s and washed in G-2 plus media, thereby removing the zona pellucida and exposing the trophectoderm. Blastocysts were subsequently incubated for 6 h in G2 media containing either no lentiviral vector (no-virus), lentiviral vectors containing a non-target shRNA and tGFP (control-virus; 1 × 10^8^ VP/ml; Sigma-Aldrich) or lentiviral vectors containing *ATP6AP2* shRNA and tGFP (knockdown-virus; 1 × 10^8^ VP/ml; sequence 5′- CCG​TGT​GGT​TTA​GTA​GAG​ATA-3’; Sigma-Aldrich) and 8ug/ml polybrene (Merck).

### 2.7 Blastocyst outgrowth

EA 5 transfected blastocysts were provided a 24 h recovery period in G2 media at 37°C in 5% O_2_, 6% CO_2_, 89% N_2_. Blastocysts were then transferred to a modified-RPMI media (RPMI-1640 supplemented with 20% FBS, 100 µM β-mercaptoethanol, 1 mM sodium pyruvate, and 5 μL/ml Penicillin-Streptomycin) for up to 120 h to examine GFP expression and embryo outgrowth. Outgrown blastocysts were imaged using the Cytation 3 microscope (Biotech, Vermont, United States) at 2.5x magnification and embryo outgrowth was measured via ImageJ software (Fuji) (Abràmoff et al., 2004).

### 2.8 Blastocyst transfer to pseudopregnant females

Male Swiss mice, 10–12 weeks of age, underwent abdominal approach vasectomy as described elsewhere (Miller et al., 2012). Post-surgery, vasectomised mice were allowed to fully recover (2–6 weeks) before mating. Swiss female mice (8–10-week-old) were then mated with vasectomised Swiss males overnight to induce pseudopregnancy. Successful mating was confirmed the next morning, via the presence of a vaginal plug (EA 1). Pseudopregnant mice were then left until EA 4, when EA 5 transfected or non-transfected embryos were ready for transfer. Blastocysts underwent non-surgical embryo transfer (NSET) via an NSET device (ParaTechs, Kentucky United States), where 10-12 viable blastocysts were transferred into each EA 4 pseudopregnant female (Green et al., 2009).

### 2.9 Sample collection and analysis of implantation and pregnancy rates

#### 2.9.1 Day 10 collections

A subset of pregnant Swiss mice (with transferred blastocysts) were monitored and weighed daily until EA 10. At this time, the pregnant mice were euthanised via cervical dislocation. The number of viable and resorbing implantation sites were recorded. The uterine horn (with implantation sites) was removed and fixed in 4% paraformaldehyde (PFA) in phosphate buffered saline (PBS) overnight at 4°C. Once fixed, two viable implantation sites (Cheng et al., 2004) were stored in 0.05% sodium azide in PBS for further immunohistochemical analyses. The remaining implantation sites were collected, snap frozen and stored at −80°C until required.

#### 2.9.2 Day 18 collections

A subset of pregnant Swiss mice (with transferred blastocysts) were monitored and weighed daily until EA 18. At this time, the pregnant mice were euthanised as described above. The number of viable and non-viable fetuses were recorded. For each viable implantation site within the uterine horn, the fetus and placenta were dissected and weighed. Each fetus along with half of its corresponding placenta was fixed as described above. The remaining half of the placenta was dissected into the junctional and labyrinth zones (Qu et al., 2014) before being snap frozen and stored at −80°C until required.

### 2.10 Immunostaining of placental tissue

#### 2.10.1 Preparation of mouse placental tissue

Paraffin-embedded EA 10 and EA 18 mouse implantation sites/placental tissues were cut into 4 µm thick sections. Sections were de-paraffinized with xylene, rehydrated with decreasing stepwise ethanol concentrations (100%–70%) and washed in running distilled water. Antigen retrieval was performed using Tris-EDTA buffer at 37°C for 15 min (Tris-Base; 0.05 mM EDTA; Triton X-100; MilliQ-Water, pH 8.0). Slides were washed in PBS with 1% Tween-20 (PBS-T) before blocking in 3% bovine serum albumin (BSA; Sigma-Aldrich) in PBS for 2 h at room temperature (RT). Tissues then underwent various immunostaining procedures as described below.

#### 2.10.2 Dual labelling immunofluorescent staining of mouse placental tissue

Following initial tissue preparation, slides were incubated with both mouse anti-cytokeratin (1:50, Dako M7018) and rabbit anti-vimentin (1:250, Abcam ab92547, Cambridge United Kingdom) primary antibodies overnight at 4°C in 1% BSA in PBS. PBS-T washes were then performed before incubation with goat anti-mouse IgG H&L Alexa Fluor^®^ 594 (1:500, Abcam ab150116) and goat anti-rabbit IgG H&L Alexa Fluor^®^ 488 (1:500, Abcam ab150077) secondary antibodies, for 1 h at RT in 1% BSA in PBS. PBS-T washes were performed before applying DAPI-ProLong Gold Antifade solution (Invitrogen) and cover slips were applied. Finally, slides were imaged at 20x magnification using an Axio Imager M2 microscope (Zeiss) and analysed using Zen imaging (Zeiss) and ImageJ software (Fuji) (Abràmoff et al., 2004).

#### 2.10.3 GFP immunofluorescent analysis of mouse placental tissue

Following initial tissue preparation, slides were probed with chicken anti-GFP primary antibody (1:2000, Abcam ab13970) overnight at 4°C in 1% BSA in PBS. PBS-T washes were performed before incubation with goat anti-chicken IgY H&L Alexa Fluor^®^ 488 (1:500, Abcam ab150173) for 1 h at RT in 1% BSA in PBS. PBS-T washes were performed before applying DAPI-ProLong Gold Antifade solution (Invitrogen) and cover slips were applied. Slides were then imaged at 10x magnification using an Axio Imager M2 microscope and analysed as above.

#### 2.10.4 Immunohistochemical analysis of (P)RR in mouse placentae

EA 18 mouse placental tissue sections were de-paraffinized and rehydrated as described above. Antigen retrieval was performed using citrate buffer at 90–100°C (0.1 M trisodium citrate in MilliQ-Water, pH 6.0) for 25 min. Slides were washed in PBS-T before blocking in 1% BSA in PBS for 1 h at RT. To reduce non-specific background staining, slides underwent a 3% H_2_O_2_ (CSA Scientific, Gillman AUS) block for 30 min at RT. After which, slides were incubated with *ATP6AP2* primary antibody (1 μg/mL; RnD Systems; AF5716, Minneapolis United States) in 1% BSA/PBS overnight at 4°C., before being incubated with a 1:300 dilution of anti-goat secondary antibody (HAF017, R&D systems) in 1% BSA/PBS for 1 h at RT. Tertiary incubation of streptavidin-biotin-horseradish peroxidase complex (ab7403, Abcam) was then carried out for 1 h at RT before immunostaining was developed by incubation in 3,3′-diaminobenzidine tetrahydrochloride solution (Metal Enhanced DAB Substrate Kit; ThermoFisher Scientific) for 10 min (Santa Cruz, sc-209686B, California United States). Cresyl violet (Sigma-Aldrich) counterstaining was applied to enhance tissue contrast before slides were cover slipped using DPX mounting solution (Sigma-Aldrich). Slides were then imaged through an Aperio slide imager at 40x magnification (Leica biosystems, Melbourne AUS).

### 2.11 Placental stereological analysis

EA 18 mouse placental tissue sections were stained with haematoxylin and eosin as previously described (Jaeger et al., 2019). The proportion of placental zones (labyrinth and junctional) compared to total placental area, were determined via ImageJ area analysis.

To determine the proportion of placental trophoblasts, fetal capillaries, and maternal blood space, placental sections underwent dual-immunofluorescent staining for vimentin and cytokeratin and imaged as previously described. The proportion of each component was then quantified using point counting on an isotropic L-36 Merz grid using the ImageJ imaging software (Abràmoff et al., 2004) (Supplementary Figure S2). Eight fields of view were collected per placental tissue at 20x magnification and uniform random sampling was employed to ensure objectivity.

The volume densities (V_d_) of placental trophoblasts, fetal capillaries, and maternal blood space were calculated using the following formula:

$$Volume\;Density;\;V_{d}=P_{a}/P_{T}$$

Where, P_a_ is the total number of points that have fallen on the component of interest and P_T_, is the total number of points applied to the image (Weibel et al., 1966; Roberts et al., 2001).

To determine the weights (C_W_) of each component the following formula was used:

$$Component\;Weight;\;C_{W}=V_{d}\;x\;total\;placental\;weight$$

The surface density (S_v_) of placental trophoblasts, fetal capillaries, and maternal blood space were calculated using the following formula:

$$Surface\;Density;\;S_{v}=2\;x\;L_{a}/L_{T}$$

Where L_a_, is the number of intercepts of the applied lines with the components of interest and L_T_, is the total length of the lines applied to the section (Weibel et al., 1966; Roberts et al., 2001).

To determine the total surface area (S_T_) of each component within the placental labyrinth zone, the following equation was used:

$$Total\;Surface\;Area;\;S_{T}=S_{v}\;x\;placental\;weight\;x\;labyrinth\;proportion$$

The mean barrier thickness (B_T_) was calculated using the following equation:

$$Barrier\;Thickness;\;B_{T}=V_{d}/S_{v}$$

Where V_d_ and S_v_, is the trophoblast volume density and trophoblast surface density, respectively (Weibel et al., 1966; Roberts et al., 2001).

To assess reproducibility, observations were repeated on sections randomly throughout the analysis. The variation observed was less than 5%.

### 2.12 Quantitative real-time polymerase chain reaction (qPCR)

RNA from HTR-8/SVneo cells was extracted using a RNeasy Mini Kit according to the manufacturer’s instructions (Qiagen, Hilden Germany). In separate experiments, RNA from mouse tissue was extracted using TRIzol (Life Technologies, California United States), following the manufacturer’s instructions. RNA integrity was validated using agarose gel electrophoresis (data not shown). Total RNA was quantified using a nanodrop ND-1000 spectrophotometer, purity was also assessed using A_260_/A_280_ and A_260_/A_230_ ratios.

Total RNA from HTR-8/SVneo cells was treated with DNase I (Qiagen) to eliminate genomic DNA before being reverse transcribed using the Superscript III reverse transcriptase kit with random hexamers (Invitrogen). Total RNA from mouse placentae was reverse transcribed using the Superscript IV reverse transcriptase kit with random hexamers (Invitrogen).

Real-time qPCR was performed in an ABI 7500 Real-Time PCR System (HTR-8/SVneo experiments) or an Applied Biosystems Quant studio 6 Flex real-time PCR system (mouse model experiments), using SYBR Green for detection. Each reaction contained 5 µL of SYBR Green PCR master mix (Life Technologies), primers (Supplementary Table S1), 10 ng cDNA and water to 10 µL. The mRNA abundance of target genes in HTR-8/SVneo cells was calculated relative to β-actin (*ACTB*) mRNA and an internal control sample (term placenta collected via elective caesarean section). The mRNA abundance of target genes in mouse placentae was calculated relative to the geomean of ACTB, beta-2 macroglobulin (B2M), and Tyrosine 3-Monooxygenase/Tryptophan 5-Monooxygenase Activation Protein Zeta (YWHAZ) and an internal control sample (mouse kidney tissue). The expression of the housekeeping genes (ACTB, B2M, and YWHAZ) did not change between treatment groups. Relative abundance was determined using the 2^−ΔΔCT^ method.

### 2.13 Protein extraction and immunoblotting

Frozen HTR-8/SVneo cell pellets were incubated on ice in RIPA protein extraction buffer (50 mM Tris-HCl, 150 mM NaCl, 1 mM EDTA, 1% NP-40, 0.5% sodium deoxycholate, 100 nM sodium orthovanadate and Complete Mini Protease Inhibitor Cocktail tablets (Roche Diagnostics, North Ryde AUS) and 1 nM PMSF. Samples were vortexed every 10 min for 30 min, then centrifuged at 13,000 rpm at 4°C for 10 min and the supernatant collected. Protein from mouse placentae was extracted using TRIzol, following the manufacturer’s instructions. Protein content was measured using a Pierce BCA Protein Assay Kit (ThermoFisher Scientific, Massachusetts United States).

Protein extracts (10 µg per well) were electrophoresed on a 4%–12% Bis-Tris protein gel (NuPAGE, ThermoFisher Scientific). Resolved proteins were transferred onto polyvinylidene difluoride (PVDF) membranes using the XCell Sure-Lock Mini-Cell electrophoresis system (BioRad, California, United States).

For HTR-8/SVneo protein samples, each membrane was blocked overnight at 4°C using 5% BSA and 5% skim milk powder in tris-buffered saline (TBS) with 0.1% Tween-20 (TBST). Membranes were then incubated at RT for 2 h with an *ATP6AP2* primary antibody (0.8 μg/mL; Abcam; ab40790), followed by a 1 h incubation with a horseradish peroxidase (HRP) conjugated goat anti-rabbit secondary antibody (0.2 μg/mL; Millipore; 12-348, Massachusetts United States). HTR-8/SVneo membranes were then stripped in 0.2 M sodium hydroxide before being re-blocked as previously described. Membranes were subsequently re-probed for ACTB (1 h; 0.04 μg/mL; Millipore; ab8227).

For mouse placental tissue, membranes were stained with ponceau S solution (0.1% ponceau, 5% acetic acid, MilliQ) for 1–5 min at RT, before being blocked for 1 h at RT in 5% skim milk in TBST. Membranes were then incubated overnight at 4°C with *ATP6AP2* primary antibody (1 μg/mL; RnD Systems; AF5716), followed by a 1 h incubation with a HRP conjugated anti-goat secondary antibody (RnD Systems).

Immunoreactivity was detected using the Amersham ECL Western blotting detection kit and imaged using the Amersham 600 imager. The density of each band was corrected for its respective loading control; *in-vitro* studies normalised to ACTB, and *in-vivo* studies normalised to total protein load. Samples were run in duplicate, and the average calculated for the final analysis.

### 2.14 Statistical analyses

All statistical analyses were performed using GraphPad Prism 8 (GraphPad Software, Inc.). Welch’s t-tests were used to analyse xCELLigence, qPCR, and Western blot data from *in vitro* HTR-8/SVneo cell experiments. Experiments were performed in technical triplicates from a minimum of three independent experiments. For the *in vivo* experiments, a Two-way ANOVA utilising Dunnett’s multiple comparisons test was used to analyse qPCR (n = 8-21), immunoblotting (n = 8-21), and blastocyst outgrowth area (n = 3-24), with samples run in technical duplicate. Fetal-placental characteristics were assessed via Kruskal–Wallis: Dunn’s multiple comparisons tests. Stereological analysis was statistically validated using Two-way ANOVA and Dunnett’s multiple comparisons, with variation between measurements assessed via Kruskal–Wallis: Dunn’s multiple comparisons test. A *p*-value of <0.05 was considered statistically significant.

## 3 Results

### 3.1 ATP6AP2 siRNA decreases trophoblast proliferation, migration, and invasion

**FIGURE 2:**
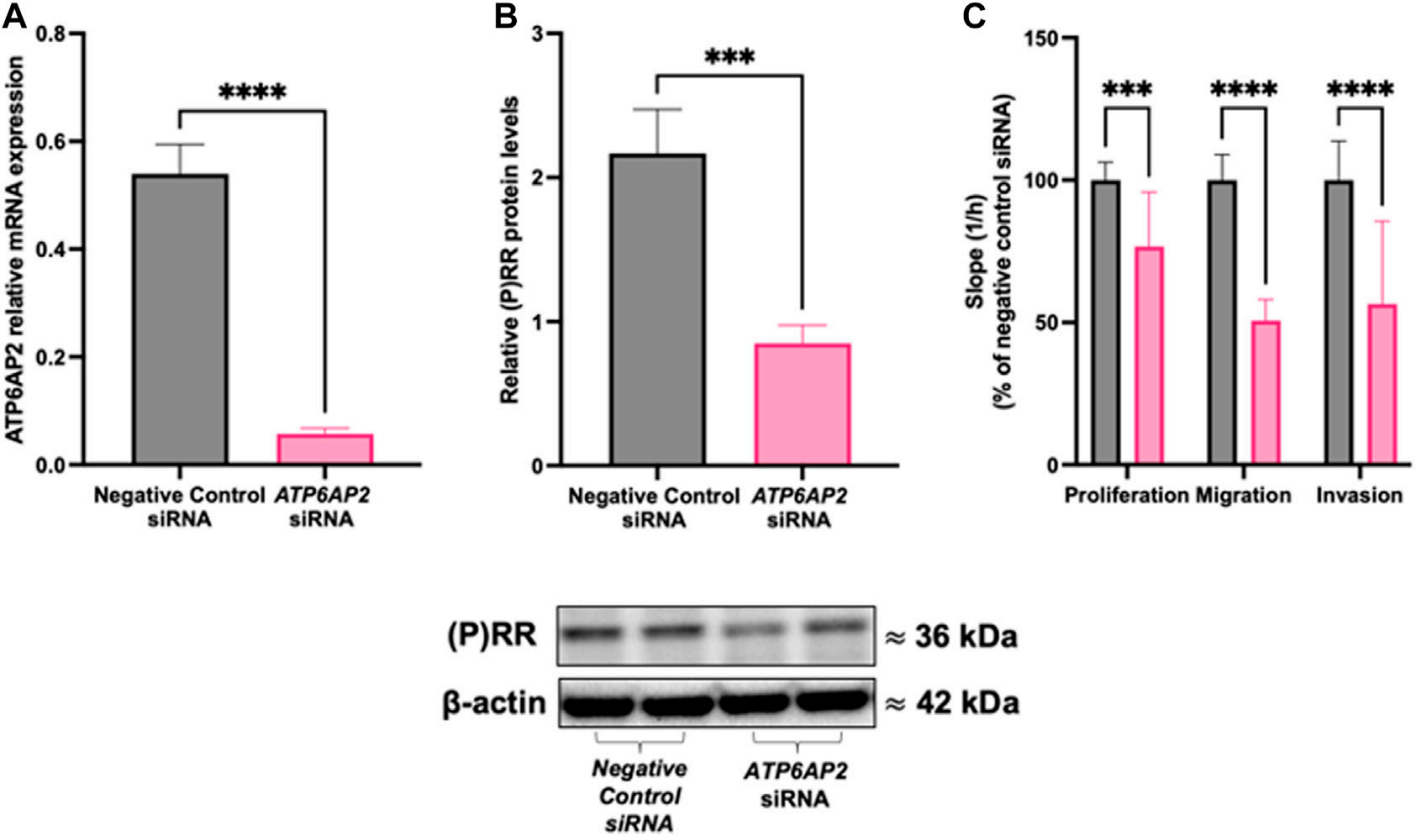
ATP6AP2 siRNA transfection in HTR-8/SVneo cells. HTR-8/SVneo cells were treated with 5 nM ATP6AP2 siRNA and cultured for 48 h. ATP6AP2 siRNA successfully decreased both **(A)** ATP6AP2 mRNA expression; and **(B)** (P)RR protein levels compared with negative control siRNA treated cells. **(C)** ATP6AP2 siRNA significantly decreased proliferation, migration, and invasion of HTR-8/SVneo cells when compared with the negative control siRNA. ***: *p* < 0.0005, ****: *p* < 0.0001 indicate significant differences between ATP6AP2 and negative control siRNA treatment groups. β-actin (ACTB) was used as a loading control in the representative immunoblot. Data were analysed by Mann-Whitney test and are presented mean +/- SEM. N = 3 experiments conducted in technical triplicates.

Treatment of HTR-8/SVneo cells with an *ATP6AP2* siRNA significantly decreased both *ATP6AP2* mRNA abundance (*p* < 0.0001; Figure 2A) and (P)RR protein levels (*p* = 0.0006; Figure 2B) compared with negative control siRNA treated cells. Treatment with an *ATP6AP2* siRNA also significantly decreased the rate of HTR-8/SVneo cell proliferation by 23% when compared with negative control siRNA treated cells (*p* = 0.0004, Figure 2C). Similarly, the rate of trophoblast migration and invasion was decreased by 44% and 43%, respectively, after *ATP6AP2* siRNA treatment when compared with negative control siRNA treated cells (*p* < 0.0001, Figure 2C).

### 3.2 ATP6AP2 lentiviral infection decreased blastocyst outgrowth area

**FIGURE 3:**
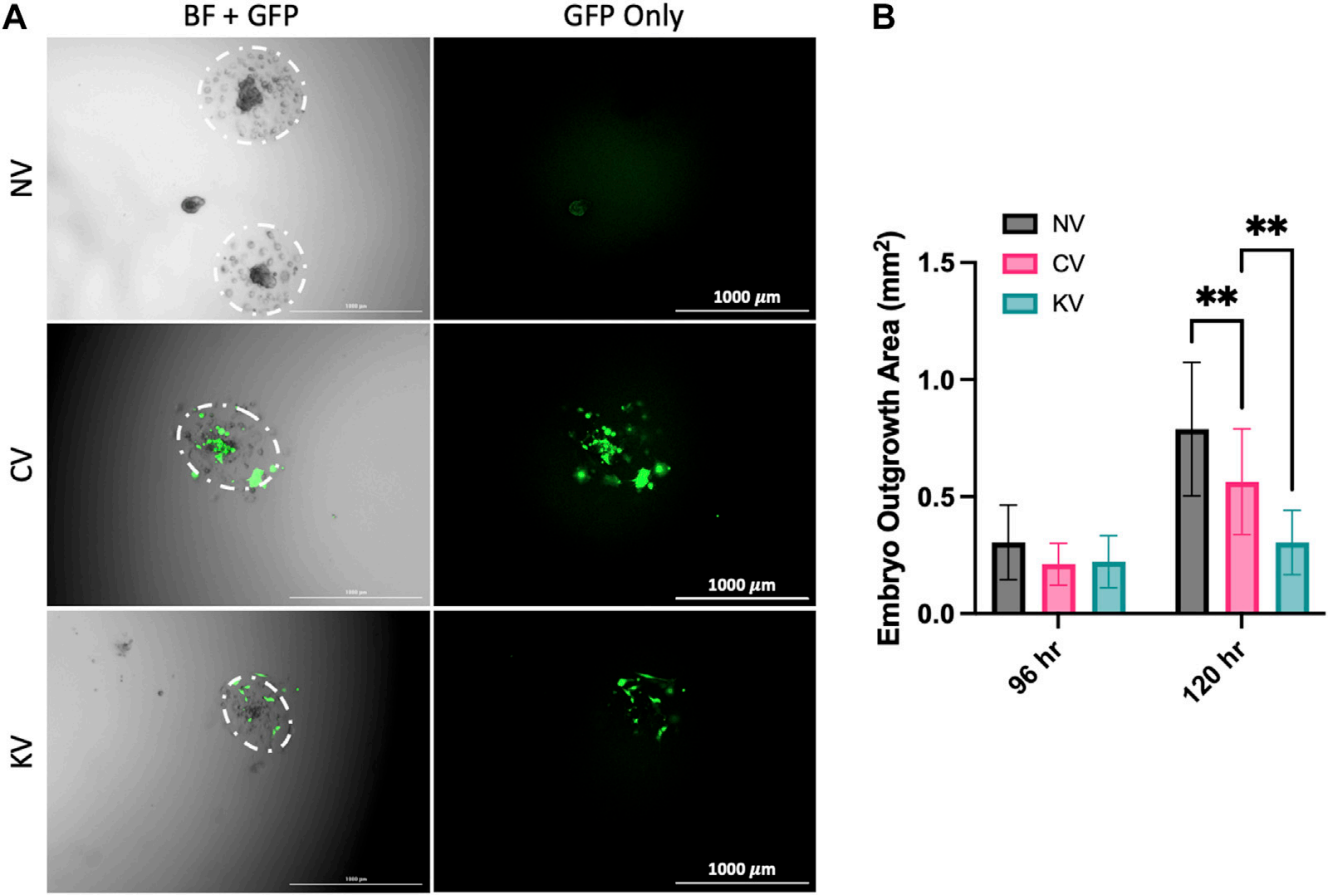
The effect of lentiviral APT6AP2 transfection on mouse blastocyst outgrowth *in vitro*. **(A)** Blastocysts were outgrown for 120 h and visual assessment of GFP was undertaken. GFP expression in blastocysts confirmed that there was successful infection of the lentivirus in the control lentivirus (CV) and the ATP6AP2 siRNA lentivirus (KV) groups. **(B)** Blastocysts were cultured for 96 or 120 h and outgrowth area was measured in cm^2^. Blastocyst outgrowth area was significantly reduced in the KV compared to the CV group. Outgrowth area was also significantly reduced in the CV group compared to the no lentiviral group (NV). BF: Bright field, GFP: Green fluorescent protein. **: *p* < 0.005, denotes significant differences between treatments. Individual blastocysts within each image and their outgrowth are outlined in white. Images were acquired at 2.5x magnification. Scale bar: 1000 μm. Data were analysed by Dunnett’s multiple comparisons test and are presented as mean +/- SEM. 96 hrs N = 15–24 blastocysts, 120 hrs N = 9 blastocysts. Biological replicates (96 hrs n = 9–13, 120 hrs n = 6 mice), total number of blastocysts analysed (96 hrs n = 15–24, 120 hrs n = 9).

Transfection of mouse blastocysts with a lentivirus containing *ATP6AP2* shRNA significantly reduced trophoblast outgrowth area measured 120 h after treatment when compared with blastocysts infected with a control lentivirus (*p* = 0.0013, Figures 3A,B). No significant change was observed between groups at the 96 h timepoints. In addition, blastocysts infected with a control lentivirus had significantly reduced outgrowth area compared with non-infected blastocysts (*p* = 0.0057, Figures 3A,B).

### 3.3 ATP6AP2 shRNA lentiviral transfection was localised to the placenta

**FIGURE 4:**
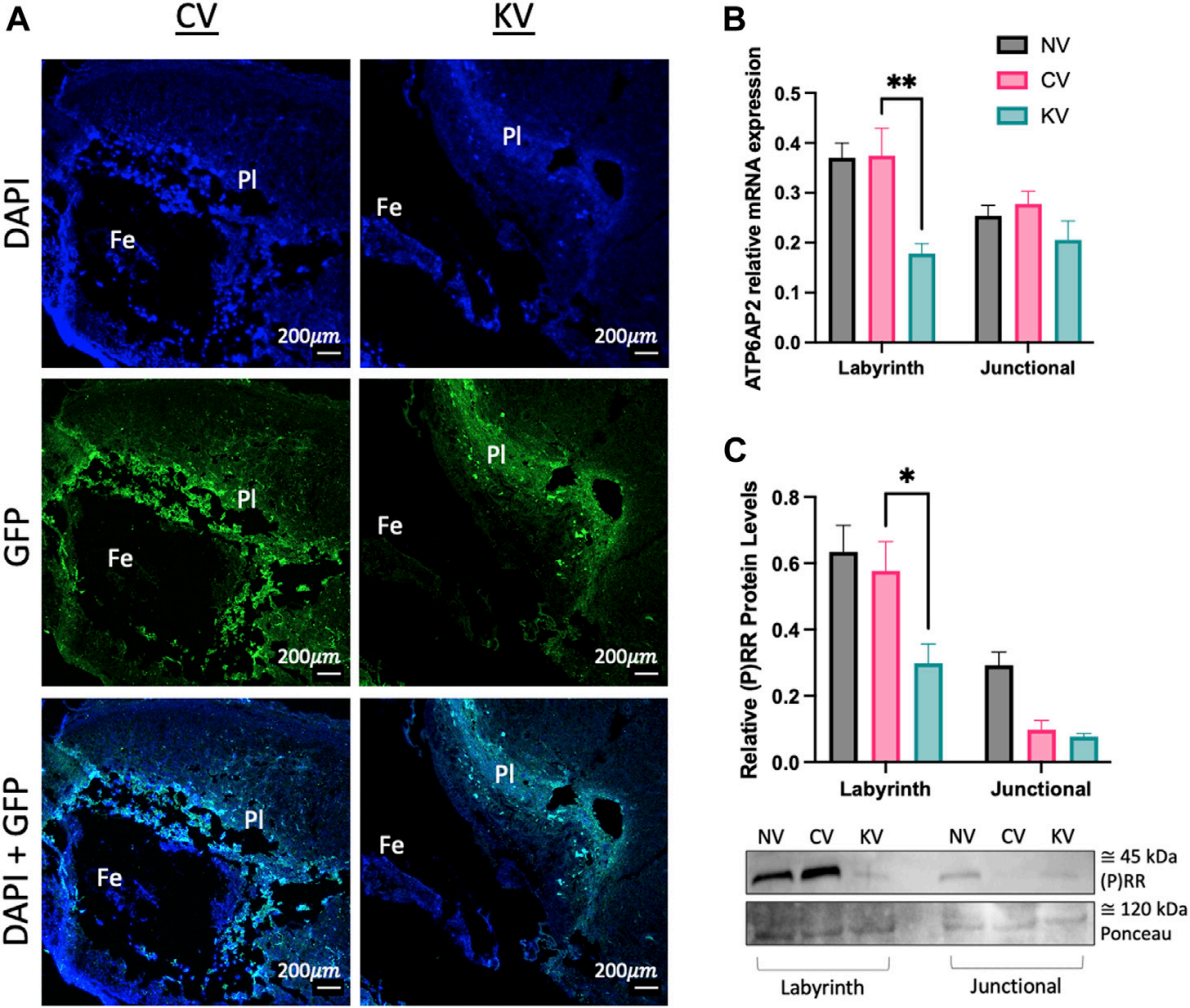
Placental (pro)renin receptor ((P)RR/ATP6AP2) expression in a mouse model of early placental (P)RR deficiency. **(A)** Green fluorescent protein (GFP) immunofluorescent staining of embryonic age (EA) 10 mouse placenta where Pl: placenta and Fe: mouse fetus. EA 18 transfected mouse placentae were separated into the labyrinth and junctional zones. **(B)** ATP6AP2 mRNA expression and **(C)** (P)RR protein levels were significantly reduced in the labyrinth zone of ATP6AP2 lentivirus infected placentae (KV) when compared with the control lentivirus infected placentae (CV). A representative immunoblot of (P)RR protein levels in mouse placenta is shown, ponceau was used as a loading control. NV: No lentivirus, CV: Control lentivirus, KV: ATP6AP2 lentivirus. *: *p* < 0.05 **: *p* < 0.005, denotes significant differences between treatments. Data are presented as mean +/- SEM. NV: n = 9 litters with 1–4 fetuses and placentae per litter (21 pups). CV: n = 6 litters with 1–5 fetuses and placentae per litter (13 pups). KV: n = 6 litters with 1–2 fetuses and placentae per litter (8 pups). Scale bar (white line) = 200 μm.

GFP expression was localised to placental tissues in *ATP6AP2* lentiviral and control lentiviral infected placentae. Importantly, no GFP expression was localised within the corresponding fetal or non-lentiviral treated placentae (Figure 4A).

(P)RR mRNA and protein levels were significantly reduced in the labyrinth zone of the placenta derived from the *ATP6AP2* lentivirus treated blastocysts when compared to the control lentivirus derived placenta (*p* = 0.002 and 0.034, respectively; Figures 4B, C). (P)RR mRNA and protein levels were similar between control lentivirus infected placentae and non-lentivirus treated placentae (Figures 4B, C). Contrastingly, no changes in (P)RR mRNA or protein levels were observed in the placental junctional zone.

### 3.4 ATP6AP2 lentiviral infection reduced placental (P)RR protein expression

**FIGURE 5:**
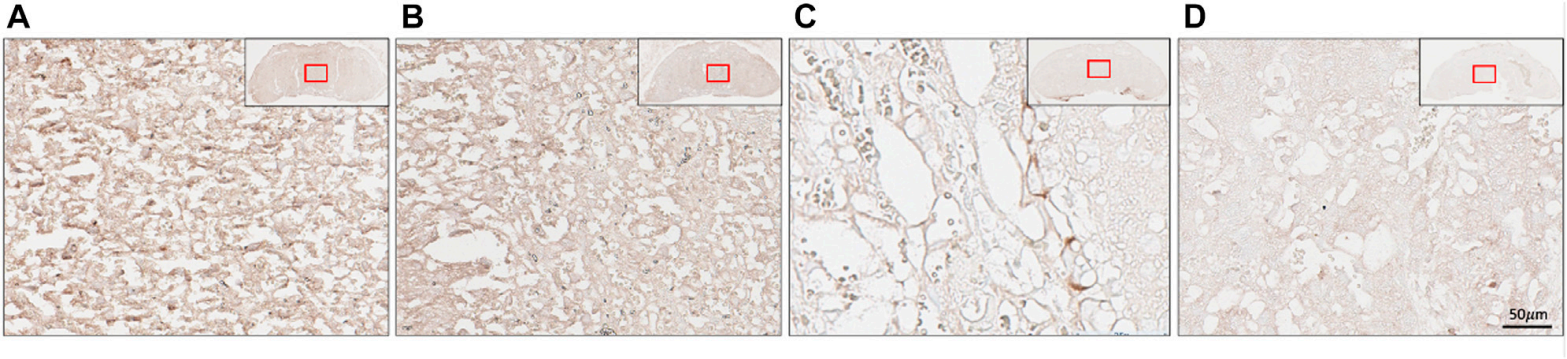
Placental localisation of the (pro)renin receptor ((P)RR). (P)RR immunohistochemical staining of embryonic age 18 mouse placental labyrinth regions. (P)RR antibody staining (brown) in **(A)** no lentivirus infected placentae; **(B)** control lentivirus infected placentae; **(C)** ATP6AP2 lentiviral infected placentae; and **(D),** no primary antibody control. Inset top right: Whole placenta pictures with red boxes highlighting regions of interest. Scale bar: 50 μM at 25x magnification.

Immunohistochemistry was performed to determine the location of (P)RR within the placenta at E18.5. The (P)RR displayed higher staining intensity around sinusoidal trophoblast giant cells (S-TGC) within the untreated (no lentivirus) blastocyst derived placenta when compared to both the control and *ATP6AP2* lentivirus infected placentae (Figure 5). Additionally, the *ATP6AP2* shRNA lentivirus derived placenta displayed less intense (P)RR antibody staining when compared with the control lentivirus infected placenta (Figure 5).

### 3.5 Placental specific ATP6AP2 lentiviral infection reduced embryo viability

Throughout gestation maternal weight was monitored and the percentage change between initial and term weight was determined (Table 1). No significant variations were observed in maternal weight between any of the three groups at EA day 10 or day 18.

**TABLE 1:** Maternal weights and pregnancy success.

	No lentivirus	Control lentivirus	ATP6AP2 shRNA lentivirus
	n = 10	n = 7	n = 7
Pregnancy Weight Change (%)			
Day 10	12.6 ± 1.624	13.3 ± 1.960	13.4 ± 1.207
Day 18	28.6 ± 1.567	34.4 ± 3.570	26.3 ± 2.037
Pregnancy Success Rates (%)			
Day 10	41.7 ± 14.9	50.0 ± 16.7	60.0 ± 16.3
Day 18	42.9 ± 13.7	38.5 ± 14.0	54.6 ± 15.8
Implantation Rates (%)			
Day 10	24.7 ± 8.6	39.1 ± 10.1	49.7 ± 9.7
Day 18	29.5 ± 9.0	22.3 ± 7.2	60.6 ± 10.4**
Reabsorption Rates (%)			
Day 10	51.7 ± 15.1	55.9 ± 17.1	60.1 ± 12.1
Day 18	73.0 ± 7.1	76.8 ± 7.8	95.2 ± 1.8*
Embryo/fetal Viability (%)			
Day 10	48.3 ± 15.1	44.1 ± 17.1	39.9 ± 12.1
Day 18	27.0 ± 7.1	23.2 ± 7.8	4.8 ± 1.8*

Data are presented as mean ± SEM., Data for each placenta treatment group were analysed by Kruskal–Wallis: Dunn’s multiple comparisons test. *Denotes a significant difference compared with the Control lentivirus infected group (*p* ≤ 0.005). Values in bold are to highlight significant changes denoted by an asterisk (*).

Implantation rate: Total number of blastocyst implantations/total number of embryos transferred per pregnancy.

Reabsorption rate: Total number of implantation reabsorptions/total number of implantation sites.

Fetal viability: Total number of viable embryos/total number of implantation sites.

The rate of pregnancy success was classified as the percentage of mice with viable implantations sites/fetuses (Table 1). No significant difference in pregnancy success rates were observed between the control and *ATP6AP2* shRNA lentiviral groups at either EA 10 or EA 18. Implantation rates were significantly increased at EA 18 between the control and *ATP6AP2* shRNA lentiviral (38.3% increase; *p* = 0.0078) groups. However, EA 18 embryo reabsorption rates were increased in the *ATP6AP2* lentiviral group (18.3% increase; *p* = 0.0377) compared to control (Table 1).

At EA 10 there was no difference in embryo viability between the control and the *ATP6AP2* lentiviral group. Conversely, at EA 18, fetal viability was found to be significantly reduced in the *ATP6AP2* lentiviral group compared with the control group (35.1% decrease; *p* = 0.0377). No changes in embryo/fetal viability were observed between the control lentivirus and the no-lentivirus treated groups at either EA 10 or EA18.

### 3.6 Reduced fetal-placental ratio in ATP6AP2 lentiviral infected placenta

Fetal and placental characteristics at E18 are outlined in Table 2. No differences in fetal head width, fetal length or fetal head width/body weight ratio were observed between any of the three groups.

**TABLE 2:** Fetal/placental characteristics.

	No lentivirus	Control lentivirus	ATP6AP2 shRNA lentivirus
	n = 21^a^	n = 13^b^	n = 8^c^
Fetal Sex (%)			
Male	61.9%	53.8%	50.0%
Female	38.1%	46.2%	50.0%
Fetal Measures (mm)			
Head Width	5.275 ± 0.2408	5.199 ± 0.1712	4.854 ± 0.3513
Body Length	24.02 ± 0.416	21.13 ± 1.737	21.21 ± 1.336
Head Width/Body Length Ratio	0.220 ± 0.010	0.256 ± 0.016	0.231 ± 0.015
Fetal/Placental Weights (g)			
Fetal Weight	0.591 ± 0.021	0.480 ± 0.048	0.491 ± 0.072
Placental Weight	0.156 ± 0.011	0.164 ± 0.025	0.147 ± 0.016
Fetal/Placental Weight Ratio	4.267 ± 0.180	4.023 ± 0.241	**2.975 ± 0.316** ^*^

^a^ n = 9 litters with 1–4 fetuses and placentae within each to a total of 21.

^b^ n = 6 litters with 1–5 fetuses and placentae within each to a total of 13.

^c^ n = 6 litters with 1–2 fetuses and placentae within each to a total of 8.

All data, except for fetal sex, are presented as mean ± SEM., Data were analysed by Kruskal–Wallis with Dunn’s multiple comparisons test (CV, control lentivirus group). *Denotes a significant difference compared with the control lentivirus infected group (*p* ≤ 0.005). Values in bold are to highlight significant changes denoted by an asterisk (*).

Fetuses and their corresponding placenta were also weighed, and the fetal/placental weight ratio calculated. This analysis revealed no significant differences in either the fetal or placental weights between the three groups. The fetal-placental ratio of the *ATP6AP2* shRNA lentivirus infected group was, however, significantly lower than the control lentivirus group (*p* = 0.0398), with no change observed compared with control lentiviral and non-viral groups.

### 3.7 ATP6AP2 lentiviral infection reduced placental trophoblast development

*ATP6AP2* shRNA lentiviral infected blastocyst derived placenta displayed significantly reduced trophoblast volume density and weight when compared with control lentiviral infected blastocyst derived placentae (both *p* < 0.0001). In contrast, maternal blood space density and component weight were increased in the placentae from the *ATP6AP2* lentiviral infected blastocysts when compared with control lentiviral infected placentae (*p* < 0.0001 and *p* = 0.0043, respectively). In addition, surface density of trophoblasts was found to be decreased and a complementary increase in maternal blood space in *ATP6AP2* lentivirus derived placentae was found when compared with control lentiviral derived placentae (both *p* < 0.0001).

Proportional assessment of placental labyrinth and junctional zones indicated an increased labyrinth but decreased junctional zone area (*p* = 0.0055) in *ATP6AP2* shRNA lentiviral infected placenta, compared with the control lentivirus group (Table 3). Taken together, with surface density estimates, there was a decreased total surface area of trophoblasts in the *ATP6AP2* lentiviral derived placentae compared to that of the control lentivirus derived placentae (*p* = 0.0002).

**TABLE 3:** Stereological assessment of placental tissue.

	No lentivirus		Control lentivirus	(P)RR shRNA lentivirus	
	*n = 21* ^a^		*N = 13* ^b^	*n = 8* ^c^	
Volume Density	TB	0.44 ± 0.02	0.48 ± 0.016	**0.26 ± 0.012** ^****^	
	FC	0.16 ± 0.012	0.17 ± 0.009	0.18 ± 0.006	
	MBS	0.42 ± 0.016	0.37 ± 0.014	**0.56 ± 0.013** ^****^	
Volume (g)	TB	0.061 ± 0.003	0.065 ± 0.005	**0.034 ± 0.003** ^****^	
	FC	0.023 ± 0.002	0.023 ± 0.002	0.024 ± 0.003	
	MBS	0.060 ± 0.005	0.052 ± 0.001	**0.074 ± 0.011** ^**^	
Surface Density (μm^2^/g)	TB	51.39 ± 2.22 **	60.56 ± 2.50	**36.64 ± 1.00** ^****^	
	FC	16.20 ± 1.50	21.33 ± 1.89	22.79 ± 1.31	
	MBS	46.81 ± 2.29	44.45 ± 1.31	**71.34 ± 0.83** ^****^	
Placental Zone Proportions	LZ	0.666 ± 0.012	0.656 ± 0.026	**0.5731 ± 0.016** ^**^	
	JZ	0.5731 ± 0.012	0.344 ± 0.026	**0.4269 ± 0.016** ^**^	
Total Surface Area (μm^2^)	TB	4.44 ± 0.19	5.50 ± 0.57	**2.81 ± 0.33** ^***^	
	FC	1.53 ± 0.17	1.93 ± 0.31	1.74 ± 0.22	
	MBS	4.43 ± 0.40	4.06 ± 0.42	5.45 ± 0.59	
Mean Barrier Thickness (μm)		90.04 ± 3.9	87.45 ± 2.21	**65.15 ± 1.21** ^*^	

^a^ n = 9 litters with 1–4 fetuses and placentae within each to a total of 21.

^b^ n = 6 litters with 1–5 fetuses and placentae within each to a total of 13.

^c^ n = 6 litters with 1–2 fetuses and placentae within each to a total of 8.

Data are presented as mean ± SEM., Data for each placenta treatment groups were analysed by Two-way ANOVA: Dunnett’s multiple comparisons test (CV, control group). *Denotes a significant difference compared with the Control lentivirus infected group (*≤ 0.05, **≤ 0.005, *** ≤0.0005, **** ≤0.0001). Values in bold are to highlight significant changes denoted by an asterisk (*).

Abbreviations: *TB*, trophoblasts; *FC*, fetal capillaries; *MBS*, maternal blood space; *LZ*, placental labyrinth zone; *JZ*, placental junctional zone.

When assessing trophoblast volume density relative to surface density, a measure of the syncytiotrophoblast barrier thickness can be estimated (Weibel et al., 1966; Roberts et al., 2001). Syncytiotrophoblast barrier thickness was significantly reduced in the *ATP6AP2* shRNA lentivirus infected placentae when compared with control lentivirus placentae (*p* = 0.0259). Trophoblast surface density was significantly lower in the no-lentivirus group when compared with control lentiviral placentae (*p* = 0.0035). Stereological analysis revealed no other differences between the no lentivirus and control lentivirus groups in the measures assessed.

## 4 Discussion

In this study, we provide a comprehensive investigation of the role of *ATP6AP2* in the development of the placenta. We have shown that by reducing *ATP6AP2* expression, both human first trimester extravillous trophoblasts (HTR8/SVneo cells) and mouse blastocysts have an impaired invasive phenotype. *In vivo,* placental specific knockdown of *ATP6AP2* decreased placental efficiency and negatively impacted embryo viability. This was associated with a reduction in placental *ATP6AP2* expression which decreased both trophoblast density and placental labyrinth zone area. While *ATP6AP2* has been shown to have roles in proliferation and invasion in non-placental cell types (Wang et al., 2019), it was unknown whether *ATP6AP2* was functionally involved in regulating placental trophoblast development. Collectively, our study shows that reductions in expression of *ATP6AP2* reduces the migration, proliferation, and invasive capacity of the placenta (Figure 2).

Since the (P)RR is an essential adaptor protein in the Wnt pathway (Ichihara and Kinouchi, 2011), the reduction in trophoblast proliferative capacity observed in this study may be due to impairment of the Wnt/β-catenin signalling pathway. The importance of Wnt/β-catenin signalling in cellular proliferation and apoptosis (Logan and Nusse, 2004) has long been observed, playing roles in breast (Yang et al., 2014), prostate (Gupta et al., 2010), and colorectal cancers (Wang et al., 2019). Recently, Wang *et al* have shown that silencing of *ATP6AP2* expression attenuates the activation of the Wnt/β-catenin signalling pathway (Wang et al., 2019), in addition to inhibiting proliferation and inducing apoptosis of human colorectal cancer cells (Wang et al., 2019; Suda et al., 2020). As well, a segment of *ATP6AP2* forms part of the V-ATPase complex (Kinouchi et al., 2010; Kinouchi et al., 2011). The V-ATPase complex is involved in lysosome function and autophagy (Mijaljica et al., 2011), which underpins cell proliferation, migration, and invasion in various cancer cells including lung adenocarcinoma (Kong et al., 2015; Ohba et al., 2020). As such, *ATP6AP2* expression is involved in lung cancer cell proliferation and progression through the regulation of autophagy (Ohba et al., 2020). Hence, *ATP6AP2* could be regulating trophoblast proliferation, migration, and invasion through the V-ATPase complex as well as the Wnt/β-catenin signalling pathway.

To confirm the role of *ATP6AP2* in placentation, we created placental-specific *ATP6AP2* deficient mice via lentiviral infection of an *ATP6AP2* shRNA specifically within the trophectoderm layer of mouse blastocysts (Figures 3, 4). Infected blastocysts were first assessed for GFP expression to ensure cells were infected prior to being transferred to recipient dams to determine the effect of *ATP6AP2* deficiency on fetal and placental development (Figure 3). GFP expression in mouse blastocysts confirmed that there was successful infection of the lentivirus in the control and *ATP6AP2* lentivirus groups (Figure 3A). Blastocysts treated with the *ATP6AP2* lentivirus exhibited reduced total outgrowth area (Figure 3B), highlighting a connection between early trophoblast proliferation, invasion, and migration, and (P)RR expression in the mouse blastocyst. Furthermore, at EA 10, GFP expression was only localised to the developing placenta and not the fetus (Figure 4A), demonstrating that the knockdown was “placental-specific”. At EA 18, we found that lentivirus containing *ATP6AP2* shRNA reduced placental labyrinth *ATP6AP2* mRNA expression by 52.4% and protein levels by 48.3% but had no effect on the expression of (P)RR in the junctional zone (Figures 4B, C). Our study therefore produced a similar degree of gene knockdown as other studies utilising this method with 70%–80% (Kubota et al., 2021; Muto et al., 2021) and 40%–60% (Wang et al., 2021) reductions in their genes of interest.

There was no effect of the ATP6AP2 shRNA lentivirus on junctional zone ATP6AP2 expression (Figures 4B, C). The question therefore arises as to why there is variation in *ATP6AP2* expression between the mouse placental junctional and labyrinth zones. Importantly, recent studies have similarly demonstrated that ATP6AP2 is abundant in the labyrinth zone of the mouse placenta (Mishima et al., 2023). Immunohistochemical assessment of (P)RR localisation suggests that (P)RR is associated with S-TGCs within the placental labyrinth zone (Figure 5). S-TGCs are a unique subtype of giant trophoblast cell localised to the smaller capillary sized sinusoids in the labyrinth, which arise from *Tpbpa*-negative progenitor cells (Hu and Cross, 2011; Pennisi et al., 2012). Future studies using co-localisation markers for s-TGCs such as cathepsin Q (Ctsq) and placental lactogen 2 (Prl3b1/PL2) (Rai and Cross, 2015) are necessary to confirm the association between S-TGCs and (P)RR expression. Previous findings showed that (P)RR is localised to the syncytiotrophoblasts and extravillous cytotrophoblasts in early gestation human placenta (Pringle et al., 2011). As such the association of the (P)RR and the trophoblast lineage requires further investigation to full elucidate its role in placental development. Additionally, *ATP6AP2* deficient placenta exhibited a reduction in the labyrinth proportional area compared to total placental area (Table 3). Hence, the reduction in labyrinth (P)RR may also be due to a decrease in total cell number in conjunction with decreased cellular *ATP6AP2* expression.

Fetal development, in part, relies on *ATP6AP2* expression with complete ablation of the *ATP6AP2* gene resulting in embryonic lethality (Sihn et al., 2010; Li et al., 2014). The (P)RR is integral to V-ATPase and the Wnt/β-catenin signalling pathways, which play key role through embryonic neuronal and renal development (Logan and Nusse, 2004; MacDonald et al., 2009; Silva-García et al., 2019). Our mouse model of early placental *ATP6AP2* deficiency parallels this outcome. At EA 18, we found that the embryos infected with a lentivirus containing *ATP6AP2* shRNA exhibited reduced viability (Table 1). In addition, EA 18 *APT6AP2* deficient placenta, displayed a reduced fetal/weight ratio (Table 2), suggesting that *ATP6AP2* is required for the functional capacity of the placenta and that deficiency impairs fetal development.

The links between a reduction in *ATP6AP2* and direct effects on placental trophoblasts is of particular interest. Utilising stereological Merz grid analysis of both the *ATP6AP2* deficient and control placenta, the volumetric distribution of trophoblasts was examined (Table 3). *ATP6AP2* deficient placenta displayed a reduction in trophoblast number and surface area. Taken together, these represent syncytiotrophoblast barrier thickness, which was also reduced in *ATP6AP2* deficient placenta. Interestingly, Morosin et al., has shown that *ATP6AP2* does not play a role in syncytialisation of term primary human trophoblast cells (Morosin et al., 2020). This suggests that a reduction in *ATP6AP2* does not directly affect syncytiotrophoblast formation but rather early trophoblast development. Interestingly, the (P)RR has been suggested to help maintain oxidative metabolism within trophoblasts (Suda et al., 2020). Considering our findings, we hypothesise that *ATP6AP2* directly effects trophoblast proliferation early in gestation leading to a reduction in total trophoblast number at term (Table 3). Hence, the number of differentiated villous cytotrophoblasts required for syncytiotrophoblast fusion (Tarrade et al., 2001) might be reduced.

The manipulation of placental *ATP6AP2* expression resulted in impaired fetal-placental outcomes, however the RAS-dependent nature of these effects is yet unknown. The RAS is involved in the embryonic emergence of hematopoietic stem/progenitor cells, implicating the RAS in embryogenesis (Julien et al., 2021). As *ATP6AP2* is involved in RAS signalling (Nguyen et al., 2002), the decreasing *ATP6AP2* expression may account for the reduction in embryo viability through the disruption of the local RAS (Rodgers and Dizerega, 2013; Julien et al., 2021). In addition, the RAS is necessary for proper placental development (Rossant and Cross, 2001; Yart et al., 2021), with a reduction in placental *ATP6AP2* decreasing placental capacity. As such, the reduction in *ATP6AP2* may be directly correlated with decreased RAS signalling and thereby decreased placental capacity.

Our study is comprised of three different models, *in vitro* human first trimester extravillous trophoblast cells, *ex vivo* blastocyst outgrowth and *in vivo* term mouse placenta. As such, this study provides a strong argument that the (P)RR is pivotal for placental development. The methodology utilised during this study has two main caveats. First, while our lentiviral transfection resulted in reduced expression of placental *ATP6AP2*, it did not result in a complete ablation of placental *ATP6AP2*. During early fetal development, the (P)RR is considered essential for fetal kidney development (Song and Yosypiv, 2011), with a complete knockout being embryonically lethal (Sihn et al., 2010; Ichihara and Yatabe, 2019). As such, changes to fetal/placental characteristics produced in this model may not be as substantial as a complete placental *ATP6AP2* knockout model. Additionally, all changes are confined within the induced aberration of *ATP6AP2* and as such, the results described in this study are limited in scope and further studies are necessary to understand the placental function of *ATP6AP2*. Second, placental specific gene manipulation can produce noteworthy variability, however all experimental comparisons are made against lentiviral control groups. Thus, experimental design has controlled for the technically strenuous nature of *in vivo* placental specific gene manipulation (Beebe et al., 2002).

We have shown for the first time that the prorenin receptor plays a significant role in growth and development of the placenta through regulation of trophoblast biology. This way, decreasing *ATP6AP2* expression in HTR-8/SVneo cells saw reduced trophoblast proliferation, migration, and invasion, confirming its integral role in these processes, which may also be relevant to cells other than trophoblasts. We have also confirmed that reducing placental (P)RR expression in a mouse model decreased total placental trophoblast cell number leading to a decrease in syncytiotrophoblast barrier thickness *in vivo* (Morosin et al., 2020). Additionally, reducing (P)RR expression reduced placental functional capacity and fetal viability. Thus, these data highlight the important role that (P)RR is playing in placental development and suggest that placentae deficient in (P)RR are likely to result in adverse fetal outcomes.

## Data Availability

The raw data supporting the conclusion of this article will be made available by the authors, without undue reservation.
