# Supplementary material for: Placental deficiency of the (pro)renin receptor ((P)RR) reduces placental development and functional capacity
Source: Front Cell Dev Biol. 2023 Aug 1;11:1212898. doi: 10.3389/fcell.2023.1212898 (PMC10427116; doi:10.3389/fcell.2023.1212898)
Supplement: Supplementary file 1 [file Presentation1.zip › Suppl. Figure 1.DOCX]

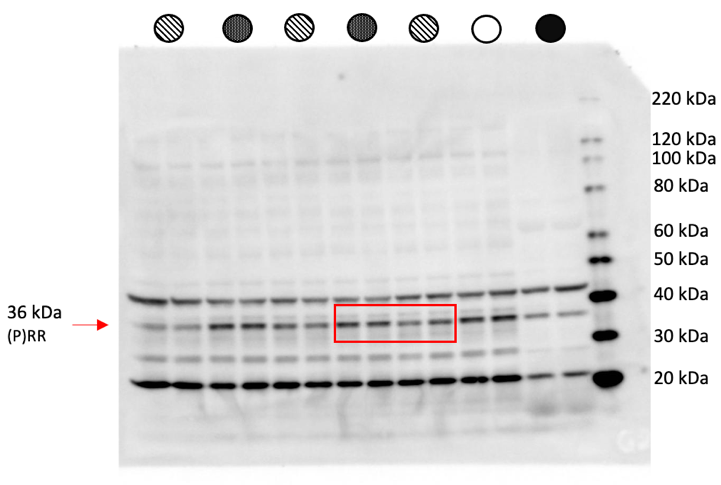

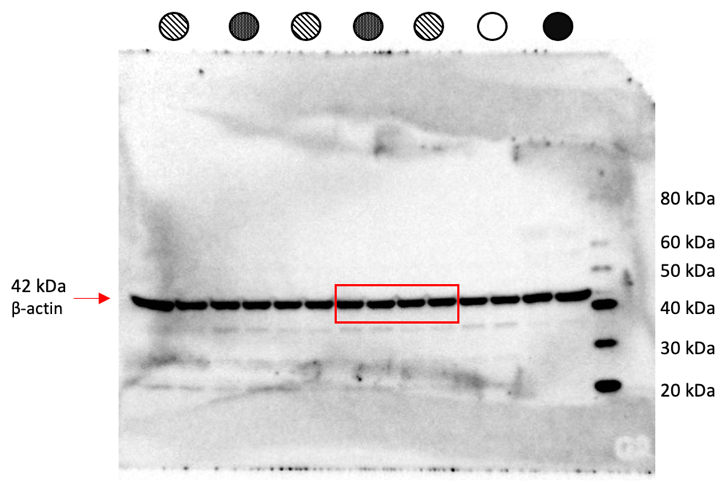

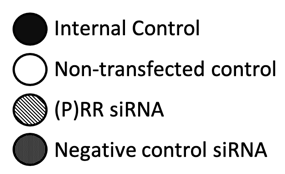


**A)**

**B)**

*Supplementary figure 1: Representative full-length immunoblot images of the (pro)renin receptor ((P)RR) and β-actin densitometry for HTR-8/SVneo cells.* In HTR-8/SVneo cells **A),** (P)RR was detected at 36kDa. Samples treated with a (P)RR siRNA knockdown, reduced (P)RR density in the 36kDa band, hence this band depicts (P)RR protein. The **B),** β-actin was detected as a clear singular band at 42kDa. **A/B** are full length blots of the representative blot in Figure 1B. The red boxes depict bands shown in the representative blot.
