# Supplementary material for: Placental deficiency of the (pro)renin receptor ((P)RR) reduces placental development and functional capacity
Source: Front Cell Dev Biol. 2023 Aug 1;11:1212898. doi: 10.3389/fcell.2023.1212898 (PMC10427116; doi:10.3389/fcell.2023.1212898)
Supplement: Supplementary file 1 [file Presentation1.zip › Suppl. Figure 2.DOCX]

*
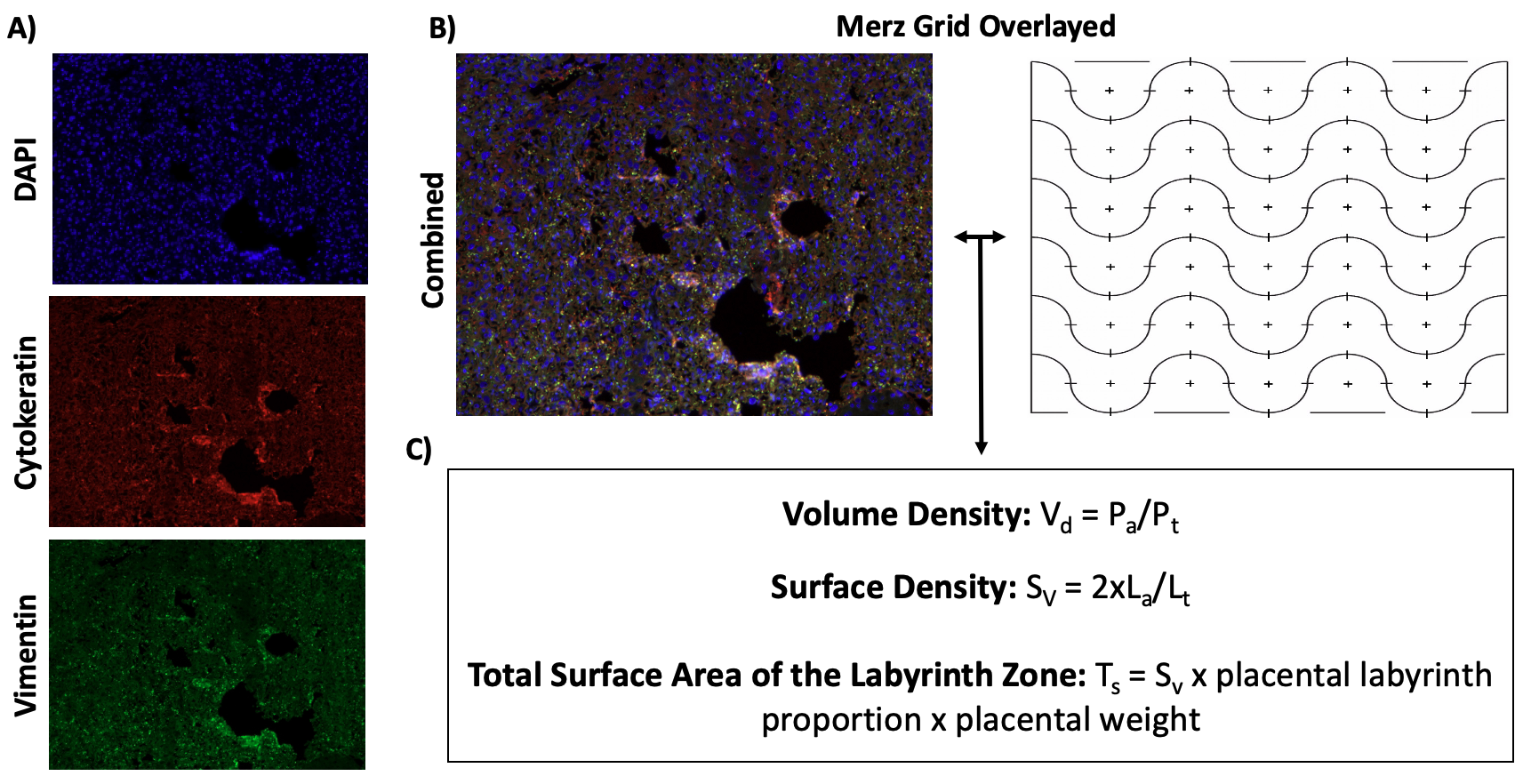
*

*Supplementary figure 2: Diagrammatic outline of stereological merz grid analysis.* In term (day 18) mouse placental sections. **A),** tissue underwent immunofluorescent staining for the following: Trophoblasts (Cytokeratin; Red), Fetal capillaries (Vimentin; Green), and a nuclear DAPI stain (Blue). After staining, placental sections were assessed by immunofluorescent microscopy where images were acquired utilising uniform random sampling. **B),** A stereological Merz Grid was applied to each image for analysis to determine volume density (V_d_), surface density (S_V_), and total surface area (T_S_) within the labyrinth of the components of interest (trophoblasts, fetal capillaries, and maternal blood space). All images were acquired at 20x magnification. Abbreviations: V_d_: Volume density. P_a_: total number of grid points per component. P_t_: total number of points applied to the image. S_V_: Surface density. L_a_: total number of line intercepts. L_t_: total length of line applied to the image. T_s_: Total surface area
