# Supplementary material for: Placental deficiency of the (pro)renin receptor ((P)RR) reduces placental development and functional capacity
Source: Front Cell Dev Biol. 2023 Aug 1;11:1212898. doi: 10.3389/fcell.2023.1212898 (PMC10427116; doi:10.3389/fcell.2023.1212898)
Supplement: Supplementary file 2 [file Table1.docx]

***Supplementary table 1: primer details for qPCR***

| **Gene** | **Species** | **GenBank Accession #** | **Primer Sequence (5' to 3')** | **Concentration (nM)** |
| --- | --- | --- | --- | --- |
| *ACTB* | Human | NM_001101 | Fwd: CGCGAGAAGATGACCCAGAT | 1000 |
|  |  |  | Rev: GAGTCCATCACGATGCCAGT |  |
| *ATP6AP2* |  | NM_005765 | Fwd: ACAATGAAGTTGACCTGCTCTTTCTTTCTG | 100 |
|  |  |  | Rev: CCTTGGCTAGATGCTTATGACGAGACA |  |
| *ATP6AP2* | Mouse | NM_027439.4 | Fwd: AAACAAGAGAACACCCAAAG | 200 |
|  |  |  | Rev: TCATATCCAGGATCCATATTCC |  |
| *ACTB* |  | NM_007393.5 | Fwd: GATGTATGAAGGCTTTGGTC | 600 |
|  |  |  | Rev: TGTGCACTTTTATTGGTCTC |  |
| *B2M* |  | NM_009735.3 | Fwd: GTATGCTATCCAGAAAACCC | 600 |
|  |  |  | Rev: CTGAAGGACATATCTGACATC |  |
| *YWHAZ* |  | NM_001253805.1 | Fwd: ACTTAACTTGTGGACATCG | 400 |
|  |  |  | Rev: GGATGACAAATGGTCTACTG |  |

Abbreviations: *Fwd;* forward sequence, *Rev;* reverse sequence, *ACTB;* β-actin, *ATP6AP2;* (pro)renin receptor, *B2M;* beta-2 macroglobulin*, YWHAZ;* Tyrosine 3-Monooxygenase/Tryptophan 5-Monooxygenase Activation Protein Zeta.
